# Detrimental Impacts of Pharmaceutical Excipient PEG400 on Gut Microbiota and Metabolome in Healthy Mice

**DOI:** 10.3390/molecules28227562

**Published:** 2023-11-13

**Authors:** Mei Zhao, Pengjiao Wang, Xiaodong Sun, Dan Yang, Shuo Zhang, Xiaoxia Meng, Min Zhang, Xiuli Gao

**Affiliations:** 1 School of Basic Medicine, Guizhou Medical University, Guiyang 550025, China; 15085969665@163.com; 2 State Key Laboratory of Functions and Applications of Medicinal Plants, School of Pharmaceutical Sciences, Guizhou Medical University, Guiyang 550025, China; wangpengjiao@gmc.edu.cn (P.W.); sunxiaodong@gmc.edu.cn (X.S.); 18311824619@163.com (D.Y.); shuozhang1015@sina.com (S.Z.); mengxiaoxia@gmc.edu.cn (X.M.); 3 Microbiology and Biochemical Pharmaceutical Engineering Research Center, Guizhou Medical University, Guiyang 550025, China; 4 Experimental Animal Center, Guizhou Medical University, Guiyang 550025, China; 5 School of Medicine and Health Management, Guizhou Medical University, Guiyang 550025, China

**Keywords:** pharmaceutical excipients, PEG400, gut microbiota, metabolomics

## Abstract

Polyethylene glycol 400 (PEG400) is a widely used pharmaceutical excipient in the field of medicine. It not only enhances the dispersion stability of the main drug but also facilitates the absorption of multiple drugs. Our previous study found that the long-term application of PEG400 as an adjuvant in traditional Chinese medicine preparations resulted in wasting and weight loss in animals, which aroused our concern. In this study, 16S rRNA high-throughput sequencing technology was used to analyze the diversity of gut microbiota, and LC-MS/MS Q-Exactive Orbtriap metabolomics technology was used to analyze the effect of PEG400 on the metabolome of healthy mice, combined with intestinal pathological analysis, aiming to investigate the effects of PEG400 on healthy mice. These results showed that PEG400 significantly altered the structure of gut microbiota, reduced the richness and diversity of intestinal flora, greatly increased the abundance of *Akkermansia muciniphila* (*A. muciniphila*), increased the proportion of Bacteroidetes to Firmicutes, and reduced the abundance of many beneficial bacteria. Moreover, PEG400 changed the characteristics of fecal metabolome in mice and induced disorders in lipid and energy metabolism, thus leading to diarrhea, weight loss, and intestinal inflammation in mice. Collectively, these findings provide new evidence for the potential effect of PEG400 ingestion on a healthy host.

## 1. Introduction

Pharmaceutical excipients are the basic materials and important components of pharmaceutical preparations, which play a very important role in the technology, dosage form, and production of pharmaceutical preparations. Pharmaceutical excipients are generally regarded as inert substances. With an in-depth understanding and research on the process of drug absorption and metabolism in vivo, it has been widely recognized that pharmaceutical excipients can impact the function of drug-metabolizing enzymes and transporters, thereby influencing the processes of drug absorption, distribution, metabolism, and excretion. Polyethylene glycol (PEG) is a polymer with excellent biocompatibility and amphiphilic properties, making it highly valuable in the field of medicine. It has a very important application in the field of medicine. It is widely used in excipients, drug carriers, and the modification of drug materials, as well as in the application of nano-preparations, liposomes, mRNA-LNP, and other new preparations [1]. PEGylation can improve the pharmacodynamic properties of drugs, enhance the targeting of drugs to tissues, and alter the fate of drugs in vivo [2,3]. Breakthroughs in PEGylation technology are propelling the field of medicine towards further advancements in precision medicine. In recent years, with the development of traditional Chinese medicine (TCM) pharmaceutical technology, new preparations such as TCM soft capsules, TCM dripping pills, and TCM injections have continued to emerge. PEG has also been widely and extensively utilized as a pharmaceutical excipient for these new TCM preparations. Our previous study demonstrated that PEG could enhance baicalin absorption and bioavailability by affecting metabolism enzymes and transporters, with PEG400 having the most pronounced effect [4].

PEG 400 is one of the very few polymers approved for injection. Due to its good solubilization and absorption-promoting effects, PEG400 is widely used in oral administration and skin and mucosa applications, especially in TCM soft capsules. It has unique advantages as it provides good dispersion and stabilization effects on the main drug while promoting intestinal drug absorption. In reality, PEG 400 is not “inert”; it not only enhances drug solubility and dispersion but also exerts various functional effects on the body. Studies have shown that PEG400 alters the systemic exposure of drugs through multiple pathways, such as drug-metabolizing enzymes and transporter functions, affecting cell membrane microenvironments and reversibly opening tight junctions [5,6,7,8,9]. Our previous study also showed that PEG400 could increase the blood concentration and tissue distribution of baicalin by inducing the activities of UGT1A8 and UGT1A9 [10,11,12]. Furthermore, we found that the long-term application of PEG400 as the matrix for TCM soft capsules can cause diarrhea and weight loss in animals, which reminds us to pay attention to the potential impact on healthy bodies. PEG400 is widely used as a dispersant and stabilizer for traditional Chinese medicine soft capsules. PEG400 is added in large quantities to traditional Chinese medicine soft capsules, but the amount is usually not indicated in the instructions. Long-term use of excipients in such medications may have adverse effects on the body and lead to changes in the drug absorption environment. As an important component of pharmaceutical preparations, it is significant to explore its influence on a healthy body, which can better guide its rational application.

Metabolome refers to the dynamic entirety of endogenous substances in organisms, and metabolites are the ultimate effect substances of gene expression. Metabolomics research can guide us in determining whether the body is healthy or diseased and help identify the root cause of diseases [13,14]. The gut microbiota is referred to as the second genome of human beings which can resist the invasion of foreign pathogens and constitute a natural biological protection barrier closely related to bodily health [15]. In recent years, intestinal microbiota has become a hot topic in research on the occurrence and development of various diseases. On the one hand, it can metabolize the contents of the gut and break them down into various metabolites. On the other hand, intestinal microorganisms can also interact with the host to effect multiple target organs and regulate homeostasis as well as disease development [16,17]. A recent human trial has shown that carboxymethyl cellulose, which is widely used in food and medicine, can cause damage to the gut microbiota, metabolome, and intestinal mucosal barrier. It increases the risk of chronic inflammatory diseases [18]. It is important to pay sufficient attention to the biological effects of pharmaceutical excipients on healthy bodies.

The aim of this study was to analyze the abundance and diversity of gut microbiota using 16S rRNA high-throughput sequencing technology, to investigate the effect of PEG on gut microbiota, to analyze the effect of PEG400 on the metabolome of healthy mice using LC-MS/MS Q-Exactive Orbtriap metabolomics technology, combined with intestinal pathological analysis, and to explore the effects of PEG400 on healthy mice. This study also aimed to provide more data support for the biological effects of PEG400 and guide the rational application.

## 2. Results

### 2.1. Impact of PEG400 on General Conditions in Mice

**Figure 1:**
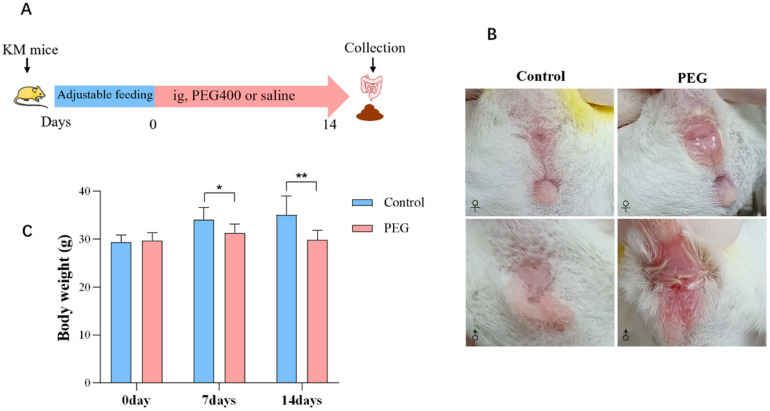
Observation of the general condition of mice. (**A**) Schematic diagram of PEG400 treatment. (**B**) Redness and swelling of anus in mice, ♀: female, ♂: male. (**C**) Mice’s body weight at days 0, 7, and 14. Data are presented as mean ± SD (*n* = 12). * *p* < 0.05, ** *p* < 0.01 compared with control group.

The experimental period lasted for 14 days, and the process is illustrated in Figure 1A. Throughout this period, the mice in the control group exhibited liveliness and activity, with smooth fur and normal bowel movements. On the other hand, the mice in the PEG group appeared quiet and weak, with disheveled hair and increased water consumption. They also experienced persistent diarrhea as well as redness and swelling around their anus (Figure 1B). Additionally, there was a significant decrease in body weight observed among the mice in the PEG group (Figure 1C).

### 2.2. Effect of PEG400 on Colon Tissue

**Figure 2:**
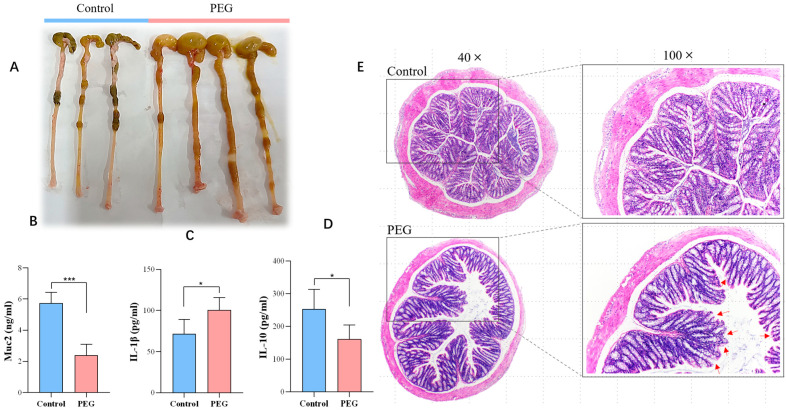
Effect of PEG400 on colon tissue. (**A**) Exterior photographs of colon tissue. (**B**–**D**) Changes in Muc2, IL-1β, and IL-10 in colonic tissue. (**E**) H&E staining image of colonic tissue; the red arrows in the figure indicate that there is incomplete intestinal mucosa, with cell exfoliation and damage to the mucus layer. Data are presented as mean ± SD (*n* = 6). * *p* < 0.05, *** *p* < 0.001 compared with control group.

The appearance of the colon of mice in the PEG group was noticeably different from that in the control group, as shown in Figure 2A. In the PEG group, the colon cavity became wider, the exterior appeared congested, the cecum was swollen and filled with gas, and the intestinal contents were thin. ELISA results (Figure 2B–D) indicated a significant decrease in Muc2 content, which is a main component of intestinal mucus; meanwhile, there was an increase in the pro-inflammatory factor IL-1β level and a marked decrease in the anti-inflammatory factor IL-10 level. As shown in Figure 2E, colon H&E staining showed that the intestinal mucosa of the control group was compact, the intestinal wall was thick, the intestinal fold structure was tight and neat, and the intestinal epithelial cell arrangement was complete. In the PEG group, the intestinal cavity became wider, the intestinal wall was thinner, the intestinal fold structure was relaxed, and the intestinal mucosa was incomplete with mild damage. The crypt spacing of the intestinal glands was widened, and goblet cells were absent and reduced in number. In summary, PEG400 has a certain impact on the integrity of intestinal mucosa and can cause mild intestinal inflammation and edema.

### 2.3. Effect of PEG400 on Gut Microbiota

**Figure 3:**
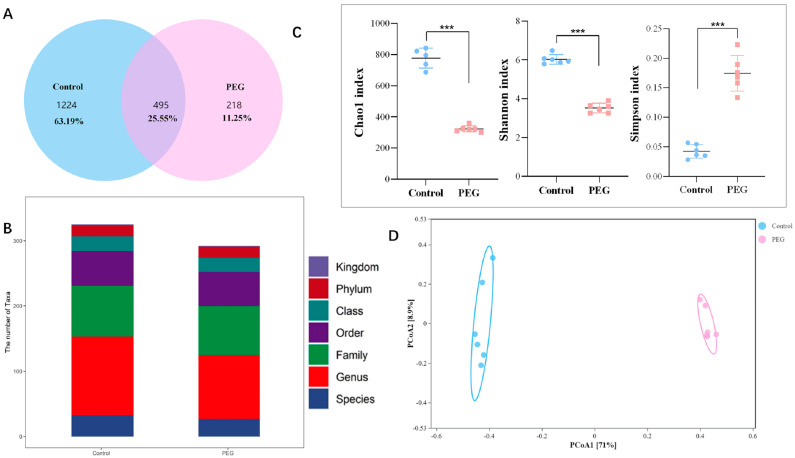
Analysis of the diversity of gut microbiota. (**A**) Venn graph of operational taxonomic units (OTUs) from the gut microbiota in the mice. (**B**) The number of taxonomic units of gut microbiota in the two groups. (**C**) The α-diversity indexes of Chao1, Shannon, and Simpson of gut microbiota in the two groups. (**D**) PCoA scatter plot of OTUs. Data are presented as mean ± SD (*n* = 6). *** *p* < 0.001 compared with control group.

Analysis of gut microbiota diversity. According to the Venn graph results, a total of 1937 operational taxonomic units (OTUs) were generated in the two groups, the total number of mutual OTUs in the control group and the PEG group was 495, the number of unique OTUs in the control group was 1224, and the number of unique OTUs in the PEG group was 218 (Figure 3A). The number of OTUs of intestinal microorganism was obviously reduced in the PEG group. There were also differences in the number of microorganisms in different taxa (Figure 3B). Chao1, Shannon, and Simpson indexes are commonly used to evaluate the alpha diversity of gut microbiota. Chao1 index reflects richness, while Shannon and Simpson indexes mainly reflect diversity. Compared with the control group, the α-diversity of gut microbiota in the PEG group was significantly decreased (Figure 3C). Further, principal coordinate analysis (PCoA) was used to conduct beta diversity analysis. As shown in Figure 3D, the bacterial composition of the control group and PEG group was obviously clustered separately. The results indicate that there were differences in intestinal microorganism composition between the two groups, and PEG400 caused changes in gut microbiota structure.

**Figure 4:**
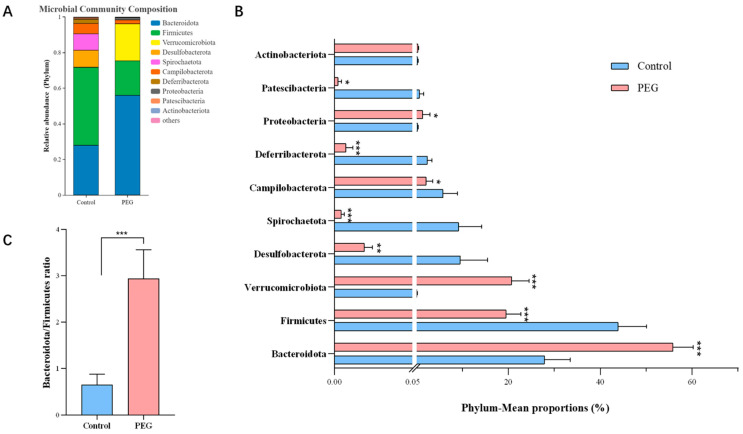
Analysis of PEG400 on gut microbiota at the phylum level. (**A**) The average percent of community abundance on phylum level in the two groups. (**B**) Plot of relative abundance statistics for the top 10 at the phylum level. (**C**) The Bacteroidetes/Firmicutes abundance ratio. Data are presented as mean ± SD (*n* = 6). * *p* < 0.05, ** *p* < 0.01, *** *p* < 0.001 compared with control group.

Analysis of PEG400 on gut microbiota at the phylum level. The effects of PEG400 on the structure of intestinal microorganisms were analyzed at different classification levels. It can be seen from Figure 4A that at the phylum level, Bacteroides, Firmicutes, Proteobacteria, Spirochaetota, and Campilobacterota were the dominant bacteria groups in the control group, accounting for more than 90%. In the PEG group, Bacteroides, Firmicutes, and Verrucomicrobiota were the dominant bacteria groups, accounting for more than 95%. Compared with the control group, the relative abundance of Bacteroidetes in the PEG group increased by 28% and that of Firmicutes decreased by 24%; the relative abundance of Verrucobacteria increased from less than 0.1% in the control group to nearly 20% (Figure 4B). PEG400 dramatically reduced the population and number of microorganisms in the gut microbiota and increased the ratio of Bacteroides to Firmicutes by 4.5 times, as shown in Figure 4C.

**Figure 5:**
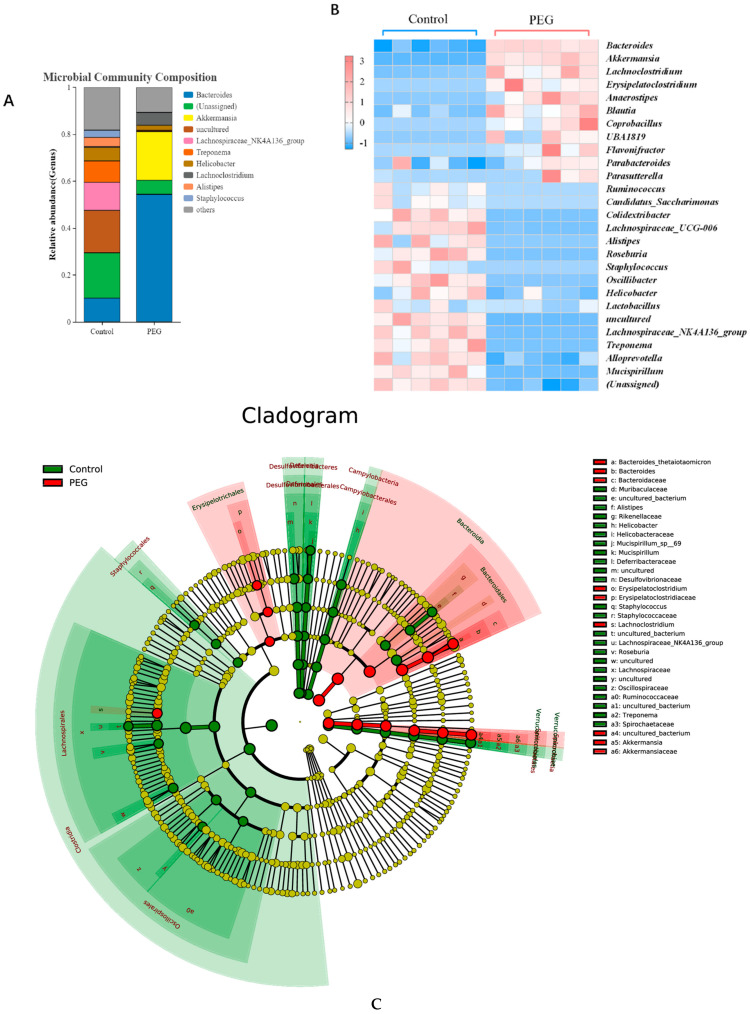
Analysis of PEG400 on gut microbiota at the genus level. (**A**) The average percent of community abundance. (**B**) Visual heat maps of microbial communities. (**C**) The LEfSe multilevel species hierarchy tree.

Analysis of PEG400 on gut microbiota at the genus level. The analysis of colony composition at the genus level showed that the dominant colony in the PEG group was relatively simple, mainly Bacteroides and *A. muciniphila* accounting for nearly 80%, while the colony composition in the control group was rich, with at least 7 species in 80% of the microorganisms (Figure 5A). A visual heatmap (Figure 5B) of microbial communities in the two groups at the genus level showed that PEG400 increased the relative abundance of *Bacteroides*, *Parabacteroides*, *A. muciniphila, Erysipelatoclostridium*, etc. The relative abundance of beneficial bacteria such as *Lachnospiraceae_NK4A136_group*, *Mucispirillum*, *Roseburia*, *Lactobacillus*, *UBA1819*, etc., was decreased. As can be seen from the LEfSe multilevel species hierarchy tree (Figure 5C), the microbial population enriched in the PEG group is small, and it mainly comes from *Bacteroides* under the phylum Bacteroides, *Erysipelatoclostridium* under the phylum Firmicutes, and *A. muciniphila* under the phylum Verrucomicrobiota. The bacteria in the control group were rich, mainly from *Bacteroides* under the phylum Bacteroides, *Lachnospiraceae* and *Oscillibacter* under the phylum Firmicutes, *Desulfovibrio* and *Campylobacter* under Proteobacteria, and *Treponema* under the phylum Spirochaetota.

### 2.4. Metabolomics Analysis

**Figure 6:**
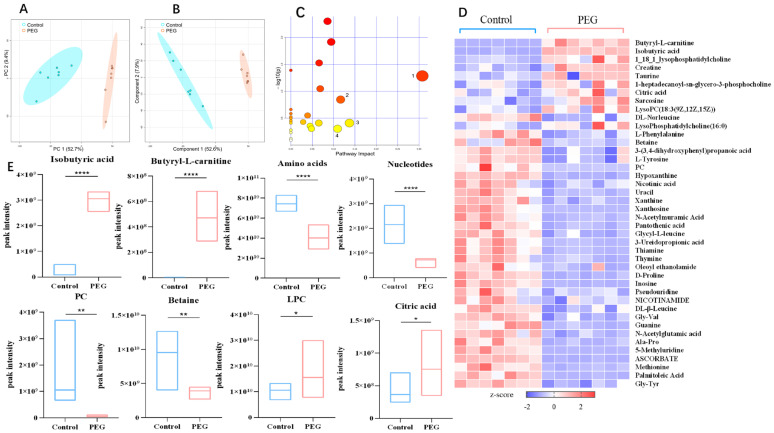
Metabolomics analysis of stools. (**A**,**B**) PCA and PLS-DA plot with the scores of the first two principal components for stools. (**C**) Metabolic pathway enrichment analysis of stools differential metabolites by MetaboAnalyst; 1, catecholamine biosynthesis, 2, phenylalanine and tyrosine metabolism,3, betaine metabolism, 4, citric acid cycle; Larger circles and darker colors indicate greater impact values. (**D**) Heatmap analysis of the 42 differential metabolites from feces; the concentration value is converted to Z-score by standardized Z-score transformation. (**E**) The box diagram for the first 8 with significant differences in metabolites. * *p* < 0.05, ** *p* < 0.01, **** *p* < 0.0001 compared with control group.

Metabolic profiling analysis. PCA and PLS-DA were used for differential analysis of fecal metabolomics data, and the analysis results are shown in Figure 6A,B. The separation of the data points between the control group and the PEG group was obvious, indicating that there were significant metabolic differences between the two groups.

Screening for differential metabolites. With variable importance for the projection (VIP) > 1 and *p* < 0.05 as the conditions, and combined with HMDB and KEGG databases to screen differential metabolites, a total of 42 differential metabolites were screened from feces, including short-chain fatty acids, butyryl-L-carnitine, PC, and degradation products LPC, such as amino acids, betaine, organic acids and derivatives, nucleotides, etc. The heatmap of differential metabolites is shown in Figure 6D. To further analyze the trend of differential metabolites between the two groups, we drew a box diagram for metabolites with significant differences; the box diagram of the variation trend of differential metabolites is shown in Figure 6E. Compared with the control group, the levels of butyryl-L-carnitine, isobutyric acid, LPC, and citric acid in the PEG group were significantly upregulated, while the levels of amino acids, PC, betaine, and nucleotides were significantly downregulated.

Metabolic pathway analysis. Differential metabolites were imported into the Metaboanalyst 5.0 database for metabolic pathway analysis. Pathways with an impact value > 0.1 were considered as those with a larger contribution value. Fecal differential metabolites were significantly enriched in catecholamine biosynthesis, betaine metabolism, phenylalanine and tyrosine metabolism, and citric acid cycle, as shown in Figure 6C.

### 2.5. Relationship between Gut Microbiota and Metabolites

**Figure 7:**
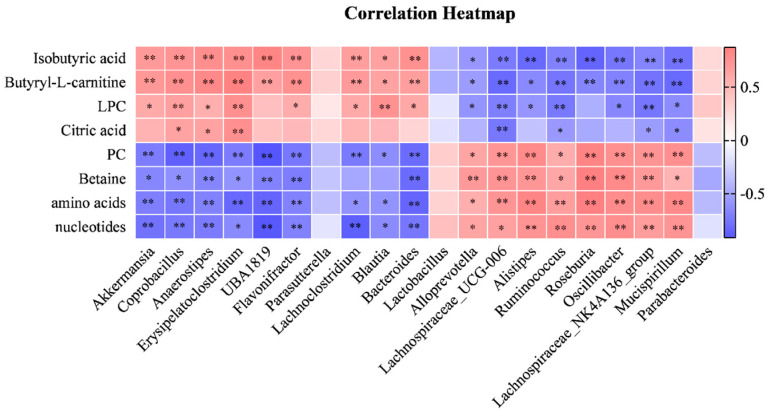
The correlation analysis of metabolites and gut microbiota. The Spearman’s correlation analysis between 8 altered metabolites and 20 altered gut microbiota, * *p* < 0.05, ** *p* < 0.01.

To further investigate the linkages between gut microbiota and fecal metabolites, we used Spearman’s correlation analysis to analyze the correlation between the fecal differential metabolites (isobutyric acid, butyryl-L-carnitine, amino acids, etc.) and the differential microbiota (*Akkermansia, Coprobacillus, Anaerostipes*, etc.) of the two groups. As shown in Figure 7, the results suggest that the differential metabolites were significantly correlated with most of the gut microbiota; some bacteria have a strong correlation with various metabolites.

## 3. Discussion

PEG400 is commonly regarded as a safe and nontoxic pharmaceutical excipient with good biocompatibility, which is widely used in the field of medicine. However, our study observed adverse effects of PEG400 on metabolomics and gut microbiota in mice, leading to diarrhea, weight loss, intestinal inflammation, and impaired intestinal mucosal integrity.

We used 16S rRNA technology to analyze the changes in gut microbiota and found significant differences between the two groups of mice. This indicates that PEG400 induced a disorder in gut microbiota, resulting in a significant reduction in richness, evenness, and diversity. It also altered the structure of the microbial community by significantly increasing the proportion of Bacteroidota and decreasing the proportion of Firmicutes. On the contrary, there was a significant increase in the proportion of Verrucomicrobiota. The gut microbiome plays a crucial role in the formation of the intestinal barrier, and disruption of its function is often associated with various intestinal diseases. Normally, Firmicutes are more abundant in the gut than Bacteroidota [19,20]. However, in the PEG group, there was a significant elevation in the relative abundances of phylum Bacteroidetes and a significant reduction in phylum Firmicutes. The addition of PEG400 significantly increased the proportion of Bacteroidota and Firmicutes, indicating an abnormal structure of the intestinal barrier, which may be one of the signs of intestinal inflammation. Bacteroides and Firmicutes are dominant microbes in the intestinal microecosystem and play a significant role in maintaining intestinal homeostasis. They are commonly referred to as “lean bacteria” and “fat bacteria,” respectively, with changes in their ratio being associated with the pathological processes of obesity, diabetes, and other metabolic diseases [21]. Bacteroidota is associated with a variety of energy metabolism functions in the body and can promote the consumption and metabolism of fats and nutrients. Bacteroidetes are beneficial bacteria only when located correctly and present in appropriate abundance [22]. The weight loss observed in mice from the PEG400 group may be related to their significant increase in abundance. It is worth noting that the relative abundance of Verrucomicrobiota in the PEG400 group increased nearly one hundred times. *A. muciniphila*, which belongs to the Verrucomicrobiota, is the only bacterial group found in human feces. It has been extensively studied as a promising strain expected to become the next generation of probiotics. [23]. It primarily colonizes the outer mucus layer of the gastrointestinal tract, utilizing mucin from the gastrointestinal tract as its energy source for growth. Simultaneously, it stimulates goblet cells to produce more mucin, promoting mucin renewal and maintaining mucus layer stability. Additionally, it can enhance tight junction expression and promote intestinal barrier integrity [24]. *A. muciniphila*, when present in appropriate abundance, has a variety of biological functions including maintaining homeostasis in the intestinal environment, inhibiting inflammation, regulating immune response, reducing the risk of certain diseases, and preventing weight gain [25,26,27]. It is a protective bacterium, and the gut can be regarded as an immune organ of the body. When the body is damaged, the gut initiates a protective mechanism by producing a large number of *A. muciniphila* to regulate the intestinal environment, immune mechanism, and inflammatory response [28]. However, the excessive proliferation of *A. muciniphila* will consume mucin excessively, causing the consumption of mucin to far exceed the generated amount. This leads to thinning of the mucus layer and destruction of intestinal mucosa, which may result in microbial invasion of intestinal epithelium and induce intestinal inflammation and other diseases. The mucus layer serves as the first line of defense against invading pathogens [29]. Muc2 is a glycoprotein synthesized by goblet cells, constituting the most abundant mucin that forms the mucus layer [30]. In this paper, the intestinal microorganisms of the PEG group exhibited a significant proliferation of *A. muciniphila*, while the level of Muc2 in the intestinal mucus layer decreased significantly. These results suggest that on one hand, PEG400 disrupts the internal environment of the intestine leading to inflammation and mucosal damage; on the other hand, it may be associated with excessive consumption of Muc2 by a large number of proliferating *A. muciniphila*. Moreover, abnormally proliferating *A. muciniphila* may be linked to initiating compensatory mechanisms for excessive self-defense.

In addition, PEG400 also reduces the abundance of beneficial bacteria such as *Roseburia*, *Lactobacillus,* and *Ruminococcus*, while increasing the abundance of harmful bacteria, like *Erysipelatoclostridium*. This may potentially contribute to intestinal inflammation and metabolic abnormalities in mice.

We performed untargeted metabolomics analysis of stool using LC-MS/MS Q-Exactive Orbtriap (Thermo Fisher Scientific, Waltham, MA, USA) and found that PEG400 altered the metabolic characteristics of mice stool. Fecal metabolomics results revealed a significant depletion of endogenous metabolites due to diarrhea in mice. First, PEG400 may interfere with phosphatidylcholine (PC) biosynthesis and affect lipid metabolism in mice, which are important components of biofilms. Additionally, lysophosphatidylcholine (LPC), a degradation product of PC, plays an essential role in regulating lipid metabolism and homeostasis and is involved in the occurrence and development of cardiovascular diseases [31,32]. The results of fecal metabolome showed that PEG400 decreased the level of PC and increased the level of LPC, its degradation product. PC is widely distributed in intestinal mucosa, and its level significantly decreases in inflammatory-mediated intestinal mucosal injury [33]. LPC binds to G-protein-coupled receptors and toll-like receptors, inducing lymphocyte and macrophage migration, promoting the production of inflammatory cytokines and increasing oxidative stress response [34]. Excessive LPC may trigger inflammation and autoimmune responses which effect the progression of metabolic diseases [35,36]. A large number of studies have also shown that when intestinal inflammation occurs, the level of inflammatory factor IL-1β in colon tissue is significantly increased, while the level of anti-inflammatory factor IL-10 is significantly decreased [37,38,39,40]. The level of pro-inflammatory factor IL-1β increases and anti-inflammatory factor IL-10 decreases prominently in the intestinal tract of mice in the PEG400 group. Further investigation is needed to determine whether this is related to the inflammatory response caused by elevated LPC levels. The literature reports that continuous gavage of 5% PEG400 for one week can cause damage to the intestinal mucosa tissue of rats, resulting in mucosal erosion and ulcer formation [41]. Our observations are consistent with this finding. Bing-Liang Ma et al. inferred that PEG400 could affect the microenvironment of cell membranes, leading to the reversible opening of tight junctions in intestinal epithelial cells and promoting the cellular bypass absorption of drugs [9]. Further investigation is warranted to determine whether PEG400 can affect biofilm function by interfering with PC biosynthesis. PEG400 possesses both good lipophilicity and hydrophilicity, exhibits high affinity for biofilms, and easily interacts with them. Therefore, the potential damage to the intestinal mucosa caused by long-term application of PEG400 should not be ignored.

PEG400 enhances betaine metabolism and reduces the level of betaine in feces. Betaine regulates cell osmotic pressure [42,43,44]. Excessive absorption of PEG400 into the body can alter the internal environment, potentially triggering self-protection mechanisms in cells that require a significant amount of betaine to prevent cell damage. Additionally, betaine plays a role in regulating lipid metabolism by participating in fat synthesis, decomposition, and transport, thereby preventing obesity and fatty liver disease caused by excessive fat accumulation. This may be one reason why the mice lost weight [45,46].

PEG400 affects phenylalanine and tyrosine metabolism. Phenylalanine is an essential amino acid in the human body which is catalyzed by phenylalanine hydroxylase (PAH) to produce tyrosine. Tyrosine is then involved in the synthesis of certain hormones or neurotransmitters. The lack of PAH in the liver leads to phenylketonuria. Maintaining stable phenylalanine and tyrosine metabolism is crucial for normal physiological function, and disruptions in their metabolism can lead to the development of metabolic diseases [47]. Tyrosine is the precursor of catecholamine synthesis, and abnormal metabolism of phenylalanine and tyrosine may hinder the biosynthesis of catecholamine neurotransmitters. This coincides with our results that PEG 400 interferes with the catecholamine biosynthesis pathway. PEG 400 increases citric acid levels in fecal matter, induces an enhanced citric acid cycle, increases energy metabolism, and accelerates the metabolism and consumption of three nutrients (sugars, lipids, and amino acids).

In addition, PEG400 increases the level of butyryl-L-carnitine in feces, which serves as an indicator of abnormal lipid and energy metabolism [48]. However, it has also been reported that butyl L-carnitine, a butyrate ester of carnitine, is known to help maintain intestinal health and prevent intestinal inflammation [49]. This outcome was unexpected. The levels of isobutyric acid, a short-chain fatty acid (SCFA), were found to be upregulated in fecal metabolites. SCFAs are metabolites produced by intestinal microorganisms and play an important role in maintaining the integrity of the intestinal barrier [50]. *A. muciniphila* and *Bacteroidetes* are also capable of metabolizing and producing SCFAs. This finding is consistent with the results of Spearman’s correlation analysis, which showed a significant positive correlation between the level of isobutyric acid and the relative abundance of *A. muciniphila*, *Bacteroidetes*, *Lachnoclostridium*, *Blautia*, etc. However, it has been observed that isobutyric acid can reduce the integrity of Caco-2 cells’ intestinal barrier and increase intestinal permeability, potentially having an opposite effect on human health. [51].

**Figure 8:**
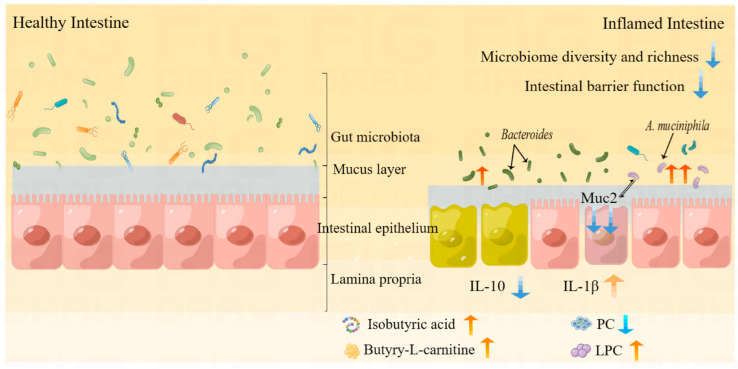
Schematic representation of intestinal injury induced by PEG400.

In our previous study on polyethylene glycol, we discovered that long-term administration of drugs containing PEG as an auxiliary material could lead to weight loss and even induce diarrhea in animals. However, this phenomenon was not observed when drugs without PEG were used alone. The main objective of this study was to investigate the adverse effects of PEG400 in mice. We have created a schematic diagram (Figure 8) to illustrate the potential adverse effects of PEG400 on the intestine. Although the dosage in this experiment is relatively high, it still holds certain reference value for understanding the potential impact of drugs containing large amounts of PEG and their long-term use on the body. Specifically, many chronic diseases require prolonged or even lifelong medication. Do auxiliary materials in such drugs alter gut microbiota and damage intestinal mucosa, thereby affecting drug absorption? This question warrants further research. Additionally, PEG400 can impact the body’s energy metabolism and reduce body weight through its metabolic byproducts and enteric microorganisms. From another perspective, PEG400 may play a role in reducing obesity and has the potential to enhance therapeutic effects when combined with anti-diabetic, anti-obesity, and other drugs for metabolic diseases.

## 4. Materials and Methods

### 4.1. Reagents

PEG400 was purchased from Beijing Solarbio Science and Technology Co., Ltd. (Beijing, China). Enzyme-linked immunosorbent assay (ELISA) kits for Interleukin-1β (IL-1β), Interleukin-10 (IL-10), and Mucoprotein 2 (Muc2) were purchased from Dongguan Enzyme Link Biotechnology Co., Ltd. (Guangdong, China). Formic acid (UPLC grade, 99% purity), methanol, and acetonitrile (UPLC grade) were obtained from Merck (Darmstadt, Germany). Unless stated, all other chemicals and reagents were of analytical grade.

### 4.2. Experimental Animals

Twenty-four Kunming mice (6–8 weeks old, SPF), half male and half female, were obtained from Hunan SJA Laboratory Animal Co., Ltd (Hunan, China. Permit Number: SCXK [Xiang] 2019-0004). Six mice were housed in each cage under controlled environmental conditions (24 ± 2 °C, 60% relative humidity), with a 12 h light/dark cycle and free access to a standard rodent diet and water. All animal experiments were approved by the experimental animal ethics committee of Guizhou Medical University (Permit Number: SYXK [Qian] 2021-0007) and performed strictly according to protocols.

After seven days of normal feeding and acclimatizing, all animals were randomly divided into two groups according to body weight. The treatment group was administered PEG 400 13 mL/kg by gavage twice a day, with an interval of at least 8 h. The control group received the same volume of saline solution. The treatment was continued for 14 days. All mice were monitored daily for living conditions. After the last administration, the mice were fasted with water for 24 h. Then, they were anesthetized and sacrificed. The mice were dissected and colonic tissues were taken. Partial colonic tissues were fixed in 4% paraformaldehyde for histological analysis, and the rest were kept in liquid nitrogen for the analysis of biochemical indexes. The mice feces samples were collected in a sterile environment and immediately frozen at −80 ℃ for subsequent 16S rRNA gene sequence and untargeted metabolomics assays.

### 4.3. Biochemical and Histological Analysis

The levels of IL-1β, IL-10, and Muc2 in colon tissue were measured using ELISA kits, following the kit’s instructions.

The fixed colonic tissues were dehydrated with graded ethanol, transparentized with xylene, embedded in paraffin, sectioned with a thickness of 4 μm by an automatic microtome, and stained with hematoxylin–eosin (H&E). The tissue sections were observed and photographed under the light microscope.

### 4.4. Gut Microbiota Composition Analysis

Gut microbiota DNA was extracted from each feces sample using a DNA extraction kit (Thermo Fisher Scientific, Waltham, MA, USA) and purity was measured using the NanoDrop One (Thermo Fisher Scientific, Waltham, MA, USA). The V3-V4 regions of 16S rRNA gene was performed for amplification using the forward primer 338F (5′-ACTCCTACGGGAGGCAGCA-3′) and the reverse primer 806R (5′-GGACTACHVGGGTWTCTAAT-3′). Primers were synthesized by Invitrogen (Invitrogen, Carlsbad, CA, USA). High-throughput sequencing was performed using the Illumina Nova6000 platform (Illumina, San Diego, CA, USA) from Guangdong Magigene Biotechnology Co., Ltd (Guangzhou, China). Sequencing libraries were generated using NEBNext^®^ Ultra™ II DNA Library Prep Kit for Illumina^®^ (New England Biolabs, Ipswich, MA, USA) following the manufacturer’s recommendations, and index codes were added.

The original data were filtered, denoised, and spliced and chimera removed. The sequences were polymerized into operational taxonomic units (OTUs) and species classification analysis was conducted using QIIME2 (https://docs.qiime2.org/2019.4/tutorials/, accessed on 26 September 2023) and the R software (v3.2.0). Chao-1, Shannon, and Simpson indexes of species were analyzed and the α-diversity was represented by principal component analysis (PCA). Clustering and thermographic analysis were performed for phylum-level and genus-level gut microflora.

### 4.5. Untargeted Metabolomic Analysis

Fifty mg of the feces samples were thawed under ice and mixed with five hundred μL of cold water–acetonitrile–methanol (1:2:2, *V*/*V*/*V*) to remove the proteins. Then, the samples were vortex-mixed to homogenize for 5 min, and ultrasonic extraction was performed in ice water for 10 min. Subsequently, the mixture was placed at −20 °C for 1h and centrifuged at 15,000 rpm, 4 °C for 15 min. Two hundred μL of the supernatant was collected and filtered through 0.22 μm filter membrane for LC-MS/MS analysis. The quality control (QC) sample was prepared with an equal contribution from each sample to ensure the stability and repeatability of the LC-MS/MS system.

Chromatography and mass spectrometry conditions. LC-MS/MS Q-Exactive Obtriap (Thermo Fisher Scientific, Waltham, MA, USA) on an Agilent (Santa Clara, CA, USA) Eclipse Plus C 18 column (2.1 mm × 100 mm, 1.8 μm) was performed for the chromatographic separation at a flow rate of 0.3 mL/min. The mobile phase consisted of 0.1% formic acid water (A) and 0.1% formic acid acetonitrile (B). The elution ratio was set as follows: 0–3 min, 5–35% B; 3–6 min, 35–55% B; 6–14 min, 55–80% B; 14–16 min, 80–100% B; 16–17 min, 100–100% B; 17–19 min, 100–5% B; 19–20 min, 5% B. The column temperature was set at 40 °C. The injection volume was set to five μL. Mass spectrometer analysis was performed automatically in both positive and negative modes (ESI^+^ and ESI^−^), respectively. The scanning range of mass spectrometry was 50–1000 *m*/*z*. The analytical conditions were as follows: ion spray voltage, +4500/−3500 V; nebulizer gas flow, 1.5 L/min; detector voltage, 1500 V; drying gas flow, 10 L/min; ion accumulation time, 20 ms; curved desolvation line (CDL) temperature, 200.0 °C.

Data processing. Firstly, the raw data were processed using the CD software (Compound Discoverer 3.3.2.31) to obtaining the data matrix consisting of normalized peak areas, retention time, and sample name. After this, analysis of the data matrix was performed to identify the differential metabolites among the different groups by SIMCA 14.1 (Umetrics, Umea, Sweden) for principal component analysis (PCA) and Partial Least Squares Discriminant Analysis (PLS-DA). In this, the significant difference metabolites were selected from the values of variable importance in the projection (VIP > 1) and combined *p* values (*p* < 0.05) and identified (the error < 10 ppm) by Kyoto Encyclopedia of genes and genomes (KEGG, http://www.kegg.ca/, accessed on 26 September 2023) and the Human Metabolome Database (HMDB, http://www.hmdb.ca/, accessed on 26 September 2023). Finally, metabolic pathway enrichment analysis was conducted for differential metabolites with [log2(FC)] > 1 or [log2(FC)] < −1 based on MetaboAnalyst 5.0 (https://www.metaboanalyst.ca, accessed on 26 September 2023). Significant metabolic pathways were those with an impact value > 0.1.

### 4.6. Statistical Analysis

All of the data were presented as the mean ± standard deviation (X ± SD). The statistical analysis between the two groups was analyzed by Student’s unpaired *t*-test using SPSS 23.0 (IBM, Chicago, IL, USA), with *p* < 0.05 taken to be significant. Correlation analysis was performed using Spearman’s correlation analysis. The analyzed data were visualized by GraphPad Prism^®^ 8 (GraphPad Software).

## 5. Conclusions

In conclusion, PEG400 significantly alters the structure of gut microbiota in mice, reduces the richness and diversity of gut microbiota, greatly increases the abundance of A. muciniphila, changes the proportion of Bacteroidetes and Firmicutes, and reduces the abundance of various beneficial bacteria. Additionally, PEG400 effects lipid metabolism and energy metabolism by inducing metabolic changes in PC, LPC, and citric acid. As a result, it causes weight loss and intestinal inflammation in mice. Our research can provide guidance for the application of PEG400 in soft capsules and serves as a reference for toxicological experimental research on medicinal excipients. Considered from another perspective, our results provide valuable insights suggesting that PEG400 may play a role in reducing obesity and has the potential to enhance therapeutic effects when combined with anti-diabetic, anti-obesity, and other drugs for metabolic diseases.

## Data Availability

Data are contained within the article.
